# How to cross the line: design principles for interdisciplinary education

**DOI:** 10.12688/mep.19693.1

**Published:** 2023-07-19

**Authors:** Jessica Oudenampsen, Enny Das, Nicole Blijlevens, Marjolein van de Pol

**Affiliations:** 1 Radboudumc department of Hematology, Radboud Universiteit, Nijmegen, Gelderland, The Netherlands; 2 Department of language and communication, Radboud Universiteit, Nijmegen, Gelderland, The Netherlands; 3 Radboudumc department of primary and community care, Radboud Universiteit, Nijmegen, Gelderland, The Netherlands

**Keywords:** Interdisciplinary Education, Higher Education, Design Based Educational Research, Medical Education, Healthcare Communication

## Abstract

**Background:** Interdisciplinary learning is gaining popularity in higher education worldwide. Currently, knowledge about how to appropriately design interdisciplinary education is still lacking. The current study presents the iterative development, pilot, and implementation of an interdisciplinary course in healthcare communication.

**Methods:** We used a design-based educational research approach in four phases to construct the program. In phase 1, we conducted a narrative review of the literature and distilled several prerequisites for interdisciplinary learning. In phase 2, we implemented two pilot courses with a focus on the content and the interdisciplinary context of the course. In research phase 3, we implemented the course during three consecutive years, with yearly evaluations of the course. In phase 4, we distilled design principles based on evaluation and reflection of the previous research phases.

**Results:** We elaborate on the various components of the design itself. Furthermore, using data from surveys, panel discussions and interviews, we reflect on the content and outcomes of the interdisciplinary course.

We propose seven evidence-informed ‘crossing the line’ design principles for future interdisciplinary education.

**Conclusions:** The developed design principles pertain to interdisciplinary education in general and transcend subject-specific boundaries. The design principles are applicable in a wide range of higher education disciplines.

## Introduction

In the rapidly changing work environment, professionals are increasingly confronted with complex problems. Patients become older and have multiple diseases that ask for integrated treatments, healthcare is becoming more expensive, sustainability issues arise in healthcare settings and management of care is becoming increasingly complex. These developments require insights and skills from different perspectives and disciplines. These changes call for more structural interdisciplinary collaboration in the work environment. In order to prepare future professionals for these increasingly complex problems it is necessary to include interdisciplinary elements into teaching programs ( Howlett *et al.*, 2016) to develop overarching skills, such as communication, technology literacy and perspective taking. Interdisciplinary education can be defined as education that takes place in the overlap between two (or more) disciplines, in which students are supported to synthesize and integrate abstract knowledge, theories and practices from both disciplines ( Boix-Mansilla *et al.*, 2000).

Interdisciplinary education generates several specific learning outcomes that are different from monodisciplinary learning outcomes, by integrating research methods, frames of reference and methodological approaches that are specific for different disciplines ( Oudenampsen *et al.*, 2022). Three categories of interdisciplinary learning outcomes can be distinguished; 1) academic and disciplinary grounding, 2) metacognitive skills such as communication, creativity and critical thinking, and 3) perspective and perception change (Unpublished paper, Oudenampsen *et al.*, 2023b).

A growing number of empirical studies illustrated that interdisciplinary learning outcomes are not easy to achieve. Previous research described the disciplinary departmental structures as most significant barriers to interdisciplinary education in higher education settings ( Newell, 2009; Welch-Devine *et al.*, 2018). Furthermore, developers and teachers continue to experience difficulties in overcoming barriers in institutional policies, procedures and resourcing ( Welch-Devine *et al.*, 2018). Although several attempts are made to encourage interdisciplinary education and interdisciplinary collaboration, many attempts fail when it comes to the implementation of interdisciplinary designs ( De Greef *et al.*, 2017; The knowledge and innovation convenant, 2020–2023).

The development of interdisciplinary education in complex contexts therefore requires a systematic approach that integrates (learning) theory and involves relevant knowledge, practices and skills from different disciplines ( Borrego *et al.*, 2014). Such a framework with designable elements for interdisciplinary learning is still lacking. Insights into specific designable elements of interdisciplinary education are needed to support educators in making informed design decisions and reach practical understanding. A design-based approach can provide these insights ( Mckenney & Reeves, 2012).

In this paper, we describe the iterative design process of an interdisciplinary course in healthcare communication, and we deducted design principles to guide the construction of interdisciplinary education, based on new theoretical and empirical knowledge. The development process to arrive at designable elements was framed from the broad paradigm of interdisciplinarity and was not limited to healthcare communication.

## Methods

### Setting

This study was conducted at the Faculty of Medical Sciences and the Faculty of Arts of the Radboud University Nijmegen, the Netherlands. It describes the design, adjustments and evaluation of an interdisciplinary course on healthcare communication.

### Context

The Radboud University features an elective program for all students. Our course was one of the first to be interdisciplinary. The course was offered to 3 ^rd^ year undergraduate medical students (MS), and 3 ^rd^ year undergraduate communication- and information sciences students (CISS). Each year, between 20 and 30 students were participating in the course.

### Design

We used an educational design-based research approach in four phases to construct the program ( Mckenney & Reeves, 2012). The design group consisted of a core of two educators (CIS and MS), one medical writer and one PhD-student. We invited undergraduate students, young professionals and educators to participate and collaborate.

Here we describe the four phases of educational design in more detail.

***Phase 1 (2018).*** During this phase, we conducted a narrative review of the literature considering two aspects: 1) a context, problems and needs analysis considering healthcare communication, and 2) the design of interdisciplinary education and the prerequisites of interdisciplinary learning.

***Phase 2 (2018–2019).*** This phase consisted of the design of two pilot courses in two design cycles. In the first design cycle, we designed a pilot course called ‘the harmonic initiative’ to test the interdisciplinary aspect of the to be developed course. Upon evaluation, the development team had regular contact with participating students and teachers. Furthermore, both the development team and the participating teachers extensively evaluated the initiative during several meetings. In the second design cycle, we piloted the content of the first version of the interdisciplinary course. Only CISS participated so we were able to reflect on the content of the course rather than on its interdisciplinary aspects. The course was evaluated by students using an online survey, and an in-classroom discussion. Moreover, we evaluated the course extensively in three team meetings after the course.

***Phase 3 (2020–2022).*** In this phase, we implemented and iterated the course three times. Box 1 provides an overview of the course. Each year, the interdisciplinary course was evaluated extensively. First, each year at the end of the course, students filled out an online survey . Second, a delegation of participating students participated in a structured panel discussion based upon the results of the online survey. Third, every student wrote down their experience with interdisciplinary learning in a reflection assignment at the end of the course. These reflections were analyzed by one author (JO). Fourth, at the end of the course, focus-group interviews (2020), individual interviews (2021) and a panel discussion (2022) were held with participating students. Analysis of these interviews was done by two research groups of which three authors (JO, MvdP and ED) were part. The results of these were published separately ( Oudenampsen *et al.*, 2023a)

**Box 1. Outline of the interdisciplinary course ‘Practice based communication innovations’**

*1. **Content of the course***

In this course, students explore how recent insights from the communication discipline can be applied in medical practice, and how these insights affect caregiver-patient conversations at the individual level. Students also learn interdisciplinary thinking and interdisciplinary collaboration, as the course is taken by both communication- and information sciences students and medical students.

In the first part of this course, students learn about important communication styles and forms that can be used in complex conversations. During several lectures, theories and practices from both Medical Sciences and Communication and Information sciences are reflected on. Teachers from both disciplines are teaching together during these lectures. Afterwards, they can experience real life conversations with simulation patients and try to apply different communication styles and linguistic aspects. After conducting the conversations, students analyze their own conversations through corpus analysis, following theories and practices of communication- and information sciences.

The second part of this course focuses on cases from professional practice concerning crisis communication; What problems arise in healthcare at the boundary of medical care and communication? Students work in groups to come up with innovative solutions for the proposed cases in a so called ‘real life action game’. Afterwards, student are challenged to dive further into theories and come up with creative and theory-driven improvements for the proposed challenges.

*2. **Structure of the course***

For seven weeks, lectures, working lectures, two conversation practicums and an interactive real life action game are held on Mondays and Wednesdays. These lectures will take approximately 3 hours each day. All lectures will take place physically.

*3. **Learning objectives***

*First part: ‘Linguistic Lab’.* The overall learning goal of the first part of the course:

You can describe the various aspects of communication, both with respect to style and form factors, which are important for therapy compliance, apply them in your own doctor-patient conversations and afterwards (quantitatively and qualitatively) analyze them. In the module Linguistic lab in the consulting room you will demonstrate the extent to which you have mastered the following learning objectives:

*Second part: ‘Real life action game and crisis communication’.* The overall goal of the second part of the course:

You can develop or improve an intervention in a team context, based on the analysis of context, stakeholders, impact and scientific literature and using the various theories from the fields of medicine and communication and information sciences. You incorporate your knowledge gained on the internship-day into an improvement report regarding one of the case solutions. With this component, you demonstrate mastery of the following learning objectives:

*4. **Assessment***

For the assessment of this course, students have to complete four different assignments:

* Individual assignment: Reflections on their own communicative footprint.

* Individual assignment: Reflections on interdisciplinary learning outcomes.

* Individual assignment: Improvement of one of the results of the real life action game, based on linguistic theories, evaluation during the real life action game and experiences during the course.

* Group assignment: scientific report based on the analysis of their own simulation-patient conversations. Students are divided into interdisciplinary groups and have to analyze three different research questions with regard to their language use and persuasive techniques.

***Phase 4 (2022).*** Each year an improvement plan was written for the next edition of the course. In phase 4, we evaluated the whole design process, reflected on the lessons learned, and we proposed design principles for interdisciplinary education in general.

**Figure 1.**
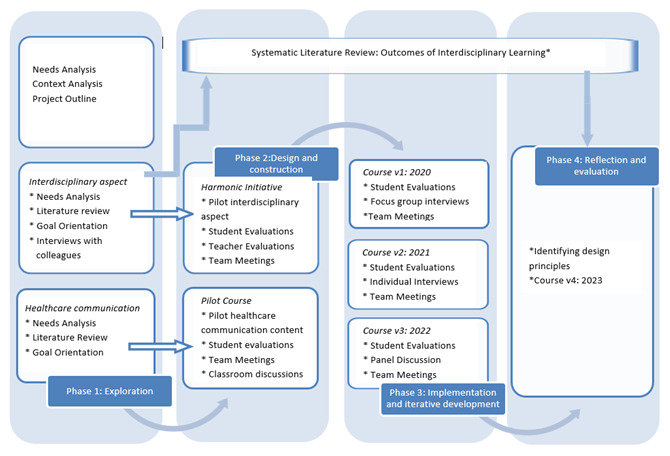
Schematic representation of the design research phases with data collection activities at each phase.

The different phases are closely linked and merge into one another, as depicted in Figure 1, which summarizes the phases and data-collection activities.

### Ethical considerations

The research participants were not part of acts that are subject to the Medical Research Involving Human Subjects Act (WMO). On this basis, the Central Medical Ethical board region Arnhem-Nijmegen the Netherlands (CMO) declares that the research does not fall under the remits of the WMO (Dossier number: CMO-020–6518). The research protocol was approved by the Research Board of the faculty of Medicine Nijmegen (Dossier number COMOS-4715217).

All participating students and teachers gave written informed consent. All data are anonymized, no identifying information is present. The research is conducted in accordance with the Declaration of Helsinki and the Netherlands Code of Conduct for Research Integrity ( Netherlands-code-conduct-research-integrity).

## Results

All data underlying the results is provided as *Underlying data* ( Oudenampsen *et al.*, 2023b).

### Phase 1: analysis and exploration

***Context, Problems and Needs Analysis Considering Healthcare Communication***. Communication education was increasingly implemented in the medical curriculum in the past years. However, while communication is par excellence a subject that is studied by different disciplines, the perspectives from these disciplines were seldom combined in education. For example, theoretical and empirical knowledge about doctor-patient communication was not yet integrated in the medical curriculum ( Framework for Medical Undergraduate Education, 2020). Conversely, while CISS gained relevant knowledge about effective communication, they had little experience in applying, testing and sharpening this knowledge in relevant practical contexts. The design team concluded that an interdisciplinary course in healthcare communication could be fruitful to innovate and enhance the knowledge and practice skills for students from both disciplines.

The design group identified two themes that could be integrated in an interdisciplinary course: communication styles during communication in the consultation room and crisis communication. We based this choice on previous student evaluations, the content of the current curricula and discussions with experts in the field.

***The design of interdisciplinary education.*** We conducted a narrative literature review of the elements in education that are necessary to achieve interdisciplinary learning. Although some relevant research was found, it became clear that overall research into interdisciplinary education and learning was scarce ( Spelt *et al.*, 2009). Studies were often explorative and no design-based recommendations were done. Even more, no examples of interdisciplinary education in healthcare communication were found. A call for empirical and design-based research into interdisciplinary learning was frequently made in existing research ( Spelt *et al.*, 2009). The research team therefore concluded that this educational design research could make an essential contribution to scientific understanding of interdisciplinary education. 

Considering the limited availability of relevant research, we identified several aspects of interdisciplinary education design from the literature. First, interdisciplinary education should focus on the integration of the perspectives of different disciplines, rather than juxtaposing perspectives of different disciplines without an effort to bring theories and practices together ( Boix-Mansilla *et al.*, 2000; Klein, 1990). Second, we included multiple collaborative elements in our course design, as literature suggests that this promotes interdisciplinary collaboration and perspective changing ( Spelt *et al.*, 2015). Third, we build on previously identified prerequisites of interdisciplinary learning ( De Greef *et al.*, 2017). We included the following prerequisites in the design of our interdisciplinary course; 1) creating a safe learning environment, 2) developing the right mindset, 3) encouraging students to communicate with each other, and 4) the teacher as a coach.

Furthermore, we adopted several conditions necessary to enable interdisciplinary learning, as identified by previous research (#2, #4, #7, #8, #9, Table 1) ( Spelt *et al.*, 2009).

**Table 1.** Design requirements based on narrative literature review.

1: Integration of disciplines
2: Use collaborative teaching elements, aimed at achieving interdisciplinarity
3: Create a safe learning environment
4: Develop the right mindset (for students and teachers)
5: Teacher as a coach
6: Encourage students to communicate with each other
7: Balance interdisciplinarity and disciplinarity
8: Reach consensus on interdisciplinarity
9: Make sure education is phased, with gradual achievement

Results of the narrative review were discussed with the development team, to finetune the problem definition and generate design requirements. The problem definition we agreed on was ‘Can we design interdisciplinary education in such a way that it encourages students to learn discipline-overarching skills? And if so, how can we do that?’. The corresponding design requirements ( Table 1) resulted in the following design proposition: ‘To design an interdisciplinary course for medicine and communication- and information sciences students, in order to teach interdisciplinary skills’.

Finally, we concluded that a systematic review of different aspects of interdisciplinary learning could be an important contribution to the knowledge about and scientific basis of interdisciplinary education design. We conducted a systematic review parallel to this project (Unpublished work, Oudenampsen *et al.*, 2023b). During this project, the outcomes of the systematic review-in-process led to iterative changes in the design of the interdisciplinary course.

### Phase 2: Design and construction

***First design cycle: Harmonic initiative***. In 2018, we ran the Harmonic initiative. On seven evenings, students and teachers came together for interactive sessions about healthcare communication and management in healthcare. Both students and teachers from the disciplines of Medicine, Humanities and Management participated and discussed for example about decision making and therapy adherence. Our goal in these sessions was to find out what topics students were interested in, how students interact with each other, and what roles teachers could take in discussions and dialogues during the sessions. Furthermore, we piloted certain pedagogical strategies that were focused on achieving collaboration and integration between disciplines. We applied the design elements as identified in design phase 1 as much as possible.

Concerning the content of the education, students stated that they were satisfied, however they suggested to better represent the different perspectives, commonalities and differences of the disciplines during the dialogues. They suggested to the teachers to take a leading and coaching role in the discussions, so that students were encouraged to communicate and discuss different perspectives with each other. Students stated they felt safe during the education, mainly due to the approachable attitude of the teachers and the space for reflection throughout the education.

Teachers felt the need to explore together even better what interdisciplinary education is and what possible outcomes of interdisciplinary education can be. In line with the students’ suggestions, teachers expressed a need to discover their common ground even further, expressing an awareness that this is needed before they can convey it to students. They also concluded it was desirable to teach with at least two teachers who both represent a different discipline, in order to encourage perspective changing. Although they gained experience in interdisciplinarity during this pilot initiative, they expressed the desire to gain more experience with it to be capable of teaching interdisciplinary.

With the Harmonic initiative, we were able to test, reinforce and validate the design principles for interdisciplinary learning as adopted in design phase 1. We learned valuable lessons, which resulted in further developed design requirements for interdisciplinary education ( Table 2).

**Table 2.** Design requirements distilled from design phase 2.

Education Design	Create space for reflection
	Intuitively appealing lectures with practical examples from all relevant disciplines
	Fit to background knowledge of both disciplines
	Focus on integration and team work in group assignments
	Provide a glossary
Teachers	Encourage dialogue and bring up possible perspectives
	Explore interdisciplinarity and common ground before the course
	Involve at least two teachers from different disciplines in design, development and implementation phase.

***Second design cycle: Pilot course for CISS***. The pilot course was constructed based upon the results from the Harmonic initiative. Great effort was invested in team development of the teachers and in the content of the course. The pilot ran in 2018 and lasted seven weeks. Because of practical issues, only CISS participated during the pilot course. The complete program consisted of lectures about linguistic aspects of healthcare communication, such as message framing and language use. Students practiced their individual (doctor-patient) communication skills during real-life conversations with simulation-patients and reported an analysis of these conversations in a research report.

We learned several lessons from this pilot ( Table 2). First, it turned out that the relevance of the lectures was not intuitively clear. Practical examples should be added to make clear to students why lectures were relevant to them and their future career.

Second, not all lectures fitted the background knowledge of CISS. Students were asked, therefore, to contribute to the final content of the course after the pilot course. We also concluded that a glossary was needed in order to familiarize students with key terminology from the other discipline. Third, attention was needed to optimize the group assignments to encourage integration and collaboration. It appeared that students independently completed a part of the group assignment, instead of integrating these aspects together as part of a team effort.

The pilot study confirmed the added value of the interdisciplinary aspect. CISS found it difficult to empathize with the perspective of healthcare professionals, as they were unfamiliar with this particular context. It was also reaffirmed that it takes two teachers from different disciplines to encourage perspective changing, both during the development and implementation of lectures.

### Phase 3: Implementation and Iterative development

During phase 3, we implemented the course during three consecutive years, with yearly evaluations of the course. In the 1 ^st^ course version (2020), we designed the final course version based on our findings of phase 2. In 2021 we executed iterative development of several course elements. In 2022, during the third version of the course, we fine-tuned course elements based on our previous findings. The lessons learned are elaborated below.

***1 ^st^ Course Version: 2020***. In the first version of the course, seven MS and 19 CISS participated. Upon evaluation, students filled out a questionnaire, wrote a reflection and participated in focus group interviews after the course. Several suggestions were made for improvement of the course. First, students suggested more teacher alignment on the practical aspects of the course. They noted that teachers from different disciplines had different perspectives on various practical issues, such as attendance requirements, handling deadlines, design of the online learning environment, and the degree of independence required of students. Second, students addressed the need for more theoretical background information, as they felt like that would help them in emphasizing the perspective of the other discipline. They suggested the implementation of introduction literature and videos about the other discipline. Third, students suggested more attention for the ways of assessment during the course, as these methods differed from the methods students were used to. Fourth, students appreciated the variety in teaching methods, such as lectures, interactive working groups and a real-life action game. Fifth, students were enthusiastic about the interdisciplinary design of the course, but continued to question the added value of interdisciplinarity. The literature review as conducted in phase 1 revealed that there was no clear overview of outcomes of interdisciplinary learning yet, and so the design team decided to focus a systematic review, that was conducted parallel to this project, on the outcomes of interdisciplinary learning.

***2 ^nd^ Course Version: 2021***. In the second version of the course 19 MS and 13 CISS participated. Upon evaluation, students filled out a questionnaire, a reflection and participated in individual interviews. We presented the preliminary results of our systematic review in the course. Students reported that this increased their awareness about possible learning outcomes, and consequently that they perceived the course as more valuable.

Students were satisfied with the amount of theory and the organization and practical aspects of the course. Now that we provided more clarity on the degree of independence required of students, it became apparent that several students were not capable of the self-efficacy and planning as expected of them. Students suggested a pre-structured study planning for students for the next version.

Another lesson we learned is that even more explicit attention to the different perspectives of students is needed to achieve real boundary crossing. As a first step in boundary crossing, students should be made explicitly aware of differences between disciplines, for which they need both the teachers and the education activities in order to aim for dialogue and discussion. As a next step, students should be able to integrate these different perspectives, for which they need education activities that aim for collaboration and integration.

***3 ^rd^ Course Version: 2022***. In the third version of the course, eight medicine students and 26 CIS students participated. Upon evaluation, students filled out a questionnaire and reflection, and students participated in a panel discussion about the course. In this version, we paid explicit attention to the difference between monodisciplinary and interdisciplinary education. Furthermore, we paid explicit attention to differences in perspectives in several parts in the course. We explicitly explained to student that we focused on two different types of learning outcomes: learning outcomes concerning healthcare communication (content) and learning outcomes on an abstract level (overarching, interdisciplinary learning). First, students reported they gained new perspectives on communication in healthcare. Second, students noted that, due to their awareness about interdisciplinary learning outcomes and perspective changing, they identified that the assessment was focused on the interdisciplinary learning outcomes. Consequently, they better understood the assessment methods that were applied in the course. Third, students mentioned that the real-life action game worked well for integrating the different perspectives and they suggested to expand the game to learn even more. In Table 3, the lessons learned in design phase 3 are summarized.

**Table 3.** Design requirements distilled from design phase 3.

Education Design	Look for theoretical depth from both disciplines
	Align practical aspects that differ between disciplines
	Pay attention to learning outcomes on the concrete level of content and on the more abstract, overarching level (interdisciplinary learning outcomes).
	Pay attention to assessment focused on interdisciplinary learning outcomes
Interdisciplinary Epistemics	Explicitly create awareness of interdisciplinary learning outcomes
	Explicitly pay attention to the integration of perspectives

### Phase 4: Evaluation and reflection

Based on the experiences in phase 2 and 3 of our educational design based research approach, the design requirements of phase 1 could be fine-tuned and several design principles and operationalizations could be distilled which can be used to design a new interdisciplinary course. Table 4 provides an overview of the developed design principles, which we divided into three overarching categories; 1) Epistemics, 2) Learning facilitators and 3) Transfer.

The categories are inspired by the framework for integrative learning environments ( Bouw *et al.*, 2021) that was published during our project. Epistemics refer to ways of knowing and how this knowledge can best be presented and structured within a curriculum. Learning facilitators concern the (physical) features of the course that support the performance of relevant tasks and facilitate achievement of relevant learning outcomes. Transfer refers to the ways in which knowledge needs to be transferred to students to achieve full potential of the education ( Bouw *et al.*, 2021).

**Table 4.** ‘Crossing the line’ Design Principles (CtL-design principles).

Design principles for interdisciplinary learning: ‘Crossing the Line’		
Epistemics	1	**Explicate interdisciplinary epistemics and outcomes** Create awareness about the added value of interdisciplinary education and learning by making explicit what interdisciplinary learning outcomes are. *Operationalization:* - Pay explicit attention for outcomes of interdisciplinary learning during development - Provide lectures about the added value of, and learning outcomes of interdisciplinary learning during the course - Give reflective assignments to students focused on interdisciplinary learning outcomes - Provide interdisciplinary assessment focused on interdisciplinary epistemics, group assignments and learning outcomes on 2 levels (i.e., overarching/metacognitive interdisciplinary skills and content).
	2	**Develop the right mindset** Reach awareness of, interest in, and consensus on interdisciplinarity among teachers. Support the development of the right (interdisciplinary) mindset for students. *Operationalization:* *Teachers* - Explore common ground between disciplines at the start of development - ‘Teach the teacher’: Expertise of teachers in interdisciplinary learning - Create alignment about shared definition and goals of interdisciplinarity *Students* -Learning activities aimed at collaboration and reflection -Promote dialogue, create awareness about differences in perspectives between disciplines -Pay attention to interdisciplinary aspects
	3	**Balance interdisciplinarity and disciplinarity** Fit to the background knowledge of students, use enough depth in disciplinary theoretical information but still create space for exploration and curiosity. *Operationalization:* * Provide intuitively appealing lectures with practical examples * Create depth in theoretical information to stimulate enthusiasm, mutual respect and interest * Align to career perspectives of different disciplines * Provide integrative learning activities * Balance well-known and new learning activities and ways of learning * Pay attention to new and well-known ways of assessment
Learning facilitators	4	**Spaces: Neutral, safe and shared** Interdisciplinary learning environments need to be safe learning environments, on ‘neutral’ or shared locations, and practical aspects need to be aligned. *Operationalization:* * Create shared online learning environments * Provide ‘neutral’ or variety of physical learning environments that represent different disciplines * Create space for reflection and evaluation * Align practical aspects during education (e.g., attendance requirements, handling deadlines, design of online learning environments, degree of independence of students)
	5	**Tutorials** Create supportive materials for students to help them understand the other disciplines theories, practices and perspectives. *Operationalization:* * Provide glossaries * Add introduction video’s * Introduce your own discipline: Students as a peer-teacher
	6	**Teacher as a coach** Guide students in seeking out perspectives they may find uncomfortable, reflect on weaknesses and guide them in thinking critical about their own discipline. *Operationalization:* * Support classroom dialogue * Dare to show vulnerability about the own discipline, or the lack of expertise with regard to the ‘other’ discipline, as a teacher
Transfer	7	**Team development and team teaching** Interdisciplinarity should be reflected in the development and teaching of the interdisciplinary education. *Operationalization:* * Teachers’ expertise in interdisciplinary learning * Co-teaching: At least two teachers (from different disciplines) per learning activity * Seek new joint learning activities, and exchange existing and integrated learning activities

 Each category consists of several subcategories with suggestions for the operationalization of a corresponding design principle.

## Discussion

This is one of the first studies to develop and evaluate an interdisciplinary course based on a systematic design-based educational approach. Iterative cycles of design and extensive evaluation and reflection offered new theoretical and empirical knowledge on the design elements important for interdisciplinary learning with impact. The chief lessons of our research are summarized in seven ‘crossing the line’ design principles for the development of interdisciplinary education ( Table 4).

The findings add to previous research in three important ways. First, previous research had already suggested various prerequisites for interdisciplinary learning that we adopted in phase 1 of our design approach. These adopted prerequisites were empirically supported by our results, thereby confirming theoretical propositions from previous research. In addition, we present tools to operationalize these prerequisites, building on evidence-based frameworks for complex learning environments ( Bouw *et al.*, 2021) The integration of previous knowledge from different fields with our research findings resulted in seven ‘crossing the line’ design principles.

Second, our study extends existing knowledge particularly on the epistemic aspect of the development of interdisciplinary learning. We observed the need to explicate interdisciplinary epistemics and outcomes for both students and teachers, in the development and implementation phases of an interdisciplinary course. Explicating the potential of interdisciplinary education provides students with awareness about how learning outcomes could differ from what they are used to. This increases conscious awareness of these skills during reflection after the course. This aligns with previous research, which described that learning elements such as reflecting, and conducting knowledge integration contributed to interdisciplinary thinking ( Spelt *et al.*, 2015).

Third, our study shows that in order to transfer the potential of interdisciplinary education to students, teachers need to develop the right mindset as well. Evidence-based benefits and learning outcomes of interdisciplinary learning need to be known by teachers, in order to convince them of the profitability of interdisciplinary education. A perceived lack of profitability could be a significant barrier in the implementation of interdisciplinary education ( Ma & Cai, 2021). Furthermore, teachers need to become aware of the norms, values, practical aspects, and goals of one’s own and other disciplines. This is necessary to understand to which degree these aspects are congruent with the organizational environment in which the interdisciplinary education is developed, and it enables to align practical aspects of both disciplines in interdisciplinary education. This positively influences the compatibility of the education, which favors interdisciplinary learning ( Cai & Lönnqvist, 2022). In addition, in our study we found empirical support for the previously suggested requirement that team development and team teaching is it is important ( De Greef *et al.*, 2017) and influences the success of implementation of interdisciplinary education ( Cai, 2017).

In the framework of Bouw *et al*. (2021), temporal (e.g., timespan and intensity) and institutional elements (e.g., disciplinary educational systems, infrastructure and social sentiment) are also considered design elements. In our research, we found several aspects concerning temporal preferences of students, i.e., differences in learning preferences and workpace of students, and difficulties with scheduling during group assignments. Moreover, we encountered many barriers in finding resources for the project and the roll-out and coordination among the various faculties. Nevertheless, we decided not to include these elements in our design principles as they are institution-dependent by their nature and, therefore, do not yield generalizable results. However, this does not imply that these aspects should not be taken into account when developing interdisciplinary learning, as such elements could hamper or facilitate interdisciplinary learning as well ( Cai & Lönnqvist, 2022).

### Strengths and limitations

Our study has some limitations. First, a known limitation of design research is its context specific nature and limited generalizability. Transfer of findings to other educational programs and systems always requires taking into account the institutional infrastructures and priorities, and the program goals and cultures. However, as interdisciplinary learning challenges these aspects by its nature, these limitations are actually strengths of interdisciplinary education as well. Moreover, we based our interdisciplinary course on discipline transcending literature that was not context specific. Even more, the clearly defined design principles and requirements are formulated discipline independent and, therefore, are of interest to the development of interdisciplinary courses in other contexts. Second, the program was initially based upon a needs analysis of a narrative review, not a systematic one. However, during phase 2,3 and 4 of our research, we conducted a systematic review in parallel. We were able to implement new theoretical insights from this systematic review in design phases 3 and 4 of our research. Our systematic review was about outcomes of interdisciplinary learning and, therefore, it is still possible that we missed publications specifically about the design and development of interdisciplinary learning.

Our study has several important strengths as well. First, the major strength of this project is that we used a systematic design based educational research approach, which is suitable for developing innovative educational programs. In reflecting on our study, we conclude that we followed the main aspects of the design research process, supporting validity of the results. A critical point may be that medicine students were not present in the pilot course in phase 2. However, as mentioned before, the pilot course was used to pilot the content of the course, rather than its interdisciplinary aspects. We consider, therefore, that the validity of the results is unaffected by this. Second, phase 1 and phase 2 of our design-based research took place before the COVID-19 pandemic. Iterative development, implementation and evaluation occurred during the COVID-19 pandemic, which might have influenced our findings, since the education took place partly digital instead of physical. However, we consider it a strength that, despite the sudden changes that had to be made in the program, evaluations were positive. Students did experience the distinctive characteristics of interdisciplinary education, such as integration, changing perspectives and interdisciplinary collaboration. Furthermore, the needed changes also challenged the development team to look even further beyond the boundaries in terms of education design. This suggests that the course itself has become even more innovative. Another important strength of this design based educational research approach was the enormously engaged research and development team, which partially countered the challenges we experienced in the project. Commitment and effort from team members is described as an important factor for the institutionalization of an educational innovation ( Cai, 2017).

## Conclusion

Based on our empirical findings, we introduced seven ‘crossing the line’ design principles for the development of interdisciplinary education. These design principles support the design of interdisciplinary education in such a way, that students are encouraged to learn discipline-overarching skills. Future research could implement and validate these design principles and provide deeper insights in the practical implementation, which, in turn, could further improve the potential of interdisciplinary education.

## Data Availability

Radboud: Unraveling interdisciplinarity: changing perspectives on interdisciplinary education, learning and learning outcomes. https://doi.org/10.34973/m7gm-8w65 ( Oudenampsen *et al.*, 2023b). Data are available under the terms of the Creative Commons Zero "No rights reserved" data waiver (CC0 1.0 Public domain dedication).
