# Supplementary material for: How to cross the line: design principles for interdisciplinary education
Source: MedEdPublish (2016). 2024 Dec 3;13:35. Originally published 2023 Jul 19. [Version 2] doi: 10.12688/mep.19693.2 (PMC11809186; doi:10.12688/mep.19693.2)
Supplement: Peer review history

# Reviewer report: Reviewer response for version 2

**DOI:** 10.21956/mep.22298.r40406

**Published:** 2025-01-16

**Authors:** Hester Smeets

**Affiliations:** 1 Zuyd University of Applied Sciences, Heerlen, Netherlands Antilles

**General comments:**

 Thank you for providing the updated version of your manuscript and for incorporating the changes. I find your approach to formulating design guidelines for interdisciplinary education through design-based research highly relevant.

 While reviewing the manuscript, I noted some questions and comments, which I have detailed in the following text.

**Introduction: **

- I find the first sentence of the paragraph "What is interdisciplinary education" difficult to follow. Wouldn't it be best to start with a simple definition? Also, why is Crossing written with a capital C? 
- To me, it seems that the second paragraph in this part should not be separate. It still concerns the difference between ID and IP education.
- In the paragraph of Constructive alignment: to me as reader I'm looking for the three steps in constructive alingment, because you speak of starts with, then 'the second step', and automatically, I'm looking for a third step, but I'm unable to find it. Could you formulate this as the first step, the second step, and the third step? 
- Did you have a research question? 
- Why do you state in the last paragraph of this introduction that the focus is not limited to communication? Why is this relevant? 

**Methods: **

- Why not start the methods with the design?
- Why is setting separate from context? 
- Phase 1: Was this literature review published? I find the methods a bit brief. Can't you provide more information in the appendix for example? So, the reader can at least know which search terms you used, and how many publications were found? Otherwise, maybe this is more your own background search, and you could include this information in the introduction, instead of your research methods? I would expect more information in this paragraph. 
- Phase 2: what topics did you evaluate on? What questions did you ask in the survey? Maybe include this information as an appendix? 
- Phase 3: what survey was used / which questions did you ask? 

**Results: **

- Phase 1: In the methods you write that you conducted a lit. review, however, in the results you write: "The design team concluded that interdisciplinary.
- course in healthcare communication could be fruitful to innovate and enhance the knowledge and practice skills for students from both disciplines.", why is there a conclusion in the results section about the lit. review? 
- You write: "Even more, no examples of interdisciplinary education in healthcare communication were found." - to me it's not clear why ID educ. is needed on healthcare communication. Why is IP education not sufficient for healthcare communication? Maybe this could be explained in the introduction. 
- You write: "Considering the limited availability of relevant research, we identified several aspects of interdisciplinary education design from the literature." --> how did you do this? How did you analyze the publications, and also very important: which publications were used?? 
- I would suggest incorporating this information in the introduction, since I now find the information provided on the methods and results too scarce to be named "literature review". 
- As I'm reading Phase 2 and Phase 3 and Phase 4 in the results, I would like more information on how you analyzed the data? Which materials were used, based on which theoretical frameworks or concepts? 
- In the tables with design requirements, there are some differences between table 1, 2 and 3, which I find confusing. In table 1, the requirements are numbered, but this is not the case in tables 2 and 3. Tables 2 and 3 have "dimensions" (e.g. education(al?) design), but table 1 does not have dimensions. Why not choose a uniform layout? 

Also, I don't see a direct link from the text to the table. How did the surveys, or evaluations, lead to the design requirements as portrayed in the tables? I would make references from the text to the concrete requirement that was based on this particular data, so the reader has an idea of how you came to those requirements. Now to me, this isn't clear. 

**Discussion:**

- Could you start with repeating the goal / aim or research question? 
- Very minor: in the second paragraph, a period is missing right after the reference. 
- You write that your research findings ass to previous research in three ways of which the third is the aspect of teachers (faculty development?). I think a lot is known about this topic in interprofessional education, but you don't seem to use this literature. Is this finding really new compared to existing literature? Can you in the discussion reflect on where the similarities are between ID and IP education, instead of focusing merely on the differences in the introduction? I think that regarding some important topics (e.g. organization, faculty development), there are more similarities than differences between those two. 

Have any limitations of the research been acknowledged?

Yes

Is the work clearly and accurately presented and does it cite the current literature?

Partly

If applicable, is the statistical analysis and its interpretation appropriate?

Not applicable

Are all the source data underlying the results available to ensure full reproducibility?

No

Is the study design appropriate and is the work technically sound?

Yes

Are the conclusions drawn adequately supported by the results?

Partly

Are sufficient details of methods and analysis provided to allow replication by others?

No

Reviewer Expertise:

Interprofessional education, assessment in interprofessional education, design-based research, qualitative research

I confirm that I have read this submission and believe that I have an appropriate level of expertise to confirm that it is of an acceptable scientific standard, however I have significant reservations, as outlined above.

# Reviewer report: Reviewer response for version 2

**DOI:** 10.21956/mep.22298.r40502

**Published:** 2025-01-08

**Authors:** Dominique Lepore

**Affiliations:** 1 Universitas Mercatorum, Rome, Italy

The authors have taken great care in revising their article based on prior feedback, and I agree with the changes they have made.

 The paper demonstrates significant improvement and addresses earlier comments effectively.

 There are a few minor suggestions that could further improve the work:

1. **Introduction: ** the introduction sets the stage for the study and could better emphasize the importance of interdisciplinary studies within healthcare. Integrating examples of recent global crises, such as the COVID-19 pandemic, would illustrate the critical relevance of this approach.
2. **Future Research Directions**: the authors might consider discussing the international dimension and the involvement of diverse stakeholders (e.g. academia, industry, government) within interdisciplinary studies. The authors could consider elaborating on the international dimension of interdisciplinary healthcare studies in future research. For instance, addressing how diverse cultural, regulatory, and economic contexts influence the integration of interdisciplinary approaches.
3. **Typographical Error**: there may be a small typographical issue in the research question section of the literature review where a question mark is missing. 

Have any limitations of the research been acknowledged?

Yes

Is the work clearly and accurately presented and does it cite the current literature?

Partly

If applicable, is the statistical analysis and its interpretation appropriate?

Not applicable

Are all the source data underlying the results available to ensure full reproducibility?

Yes

Is the study design appropriate and is the work technically sound?

Yes

Are the conclusions drawn adequately supported by the results?

Yes

Are sufficient details of methods and analysis provided to allow replication by others?

Yes

Reviewer Expertise:

applied economics; innovation ecosystems; digital technologies.

I confirm that I have read this submission and believe that I have an appropriate level of expertise to confirm that it is of an acceptable scientific standard.

# Reviewer report: Reviewer response for version 2

**DOI:** 10.21956/mep.22298.r40407

**Published:** 2025-01-03

**Authors:** Catherine Witkop

**Affiliations:** 1 Associate Dean for Medical Education, Uniformed Services University, BETHESDA, USA

I have no further comments to make.  The authors addressed all of my concerns and comments.

Have any limitations of the research been acknowledged?

Yes

Is the work clearly and accurately presented and does it cite the current literature?

Yes

If applicable, is the statistical analysis and its interpretation appropriate?

Not applicable

Are all the source data underlying the results available to ensure full reproducibility?

Yes

Is the study design appropriate and is the work technically sound?

Yes

Are the conclusions drawn adequately supported by the results?

Yes

Are sufficient details of methods and analysis provided to allow replication by others?

Yes

Reviewer Expertise:

I have a PhD in Health Professions Education and have experience in curricular design and evaluation. My areas of research are in patient-provider communication and shared decision making.

I confirm that I have read this submission and believe that I have an appropriate level of expertise to confirm that it is of an acceptable scientific standard.

# Reviewer report: Reviewer response for version 2

**DOI:** 10.21956/mep.22298.r40500

**Published:** 2024-12-26

**Authors:** Andreta Slavinska

**Affiliations:** 1 Rîga Stradiòð University, Rîga, Latvia

The article explores design principles for interdisciplinary education, offering practical recommendations and methodologies to enhance student engagement and learning experiences across disciplines. The study utilizes qualitative data analysis, based on the implementation and evaluation of pilot courses over several years, and provides clearly defined recommendations grounded in extensive experience.

- The article is well-structured and clear, with a detailed introduction and logical argumentation. The references are up-to-date and relevant to support the study's objectives and conclusions.
- The study uses a design-based research approach, which is effective for developing interdisciplinary education principles. Each phase and method used is well-detailed.
- The methodology section is well-documented, providing enough information for replication or adaptation of the study in similar contexts.
- The study relies on qualitative data analysis (interviews, discussions, and surveys), so statistical analysis is not relevant in this context.
- The article acknowledges that the generalization of conclusions is limited to specific educational environments. Suggestions for future research are provided to expand the applicability of these principles.
- The conclusions logically derive from the data collected and offer clear and practical recommendations for improving education.
- Overall, the article is well-written and makes a significant contribution to the research on interdisciplinary education. The authors have conducted a thorough study, and only minor improvements would further enhance its scientific value.
- Additional Suggestions for Improvement
- Add a short reflection on the importance of professional development and training for educators implementing interdisciplinary approaches.  

Have any limitations of the research been acknowledged?

Yes

Is the work clearly and accurately presented and does it cite the current literature?

Yes

If applicable, is the statistical analysis and its interpretation appropriate?

Not applicable

Are all the source data underlying the results available to ensure full reproducibility?

Yes

Is the study design appropriate and is the work technically sound?

Yes

Are the conclusions drawn adequately supported by the results?

Yes

Are sufficient details of methods and analysis provided to allow replication by others?

Yes

Reviewer Expertise:

Simulation based  medical education.

I confirm that I have read this submission and believe that I have an appropriate level of expertise to confirm that it is of an acceptable scientific standard.

# Reviewer report: Reviewer response for version 1

**DOI:** 10.21956/mep.21094.r34511

**Published:** 2023-09-15

**Authors:** Catherine Witkop

**Affiliations:** 1 Associate Dean for Medical Education, Uniformed Services University, BETHESDA, USA

The authors utilized a design-based educational research approach to construct an interdisciplinary course in healthcare communications and share the iterative design process in this article. They used design principles to guide the construction, based on new theoretical and empirical knowledge. The work is clearly and accurately presented and cites some of the current literature, although the supporting references are not comprehensive.

 Overall, the Methodology is sound and the Results section and Discussion are clearly written. The comments below are meant as considerations to help improve the manuscript and provide some clarity for the reader:

1. Page 3, second paragraph, the authors state: “ *Three categories of interdisciplinary learning outcomes can be distinguished: “(1) academic and disciplinary grounding, (2) metacognitive skills such as communication, creativity and critical thinking, and (3) perspective and perception change*” and cite an unpublished paper by one of the authors (Oudenampsen et al, 2023b). Especially given the unpublished nature of the citation, would recommend a little more detail about these three categories. How were they conceived? If the unpublished paper contains research around these categories, would recommend more information about this research.
2. The authors clearly state their objective in the last paragraph of the Introduction. This flows well from the background provided in the Introduction and helps frame the paper that follows.
3. Page 3, Context: It might be helpful to provide more details about the undergraduate communication and information sciences (CISS) program. Because this program may not be familiar to many readers, it would be helpful to better understand the context of this study and how it may be similar or not to another context.
4. Page 3, Design: Would recommend more comprehensively addressing reflexivity, by providing, at the least, more details about the design group. 
5. Page 3, Phase 2: The authors state: “ *In the second design cycle, we piloted the content of the first version of the interdisciplinary course. Only CISS participated . . .*”. It is not clear why this decision was made, given that the course objectives were likely centered around the interdisciplinary nature of the course—how were the authors able to “evaluate” the interdisciplinary objectives when it was only a single discipline who was participating?
6. Page 4, Box 1: Why did the authors not include the course learning objectives in Box 1? Especially given that the authors did not find clearly delineated outcomes of interdisciplinary learning during the literature review in Phase 1, it would be important for the reader to understand what the design team developed for learning objectives.
7. Page 7, It seems that during Phase 2, changes were made to the content of the course based on the CISS students. Is this the case and, if so, was the design team concerned about this biasing the ultimate content toward what is needed for CISS students? While the authors state that the “ *pilot study confirmed the added value of the interdisciplinary aspect*”, it seems important to acknowledge this as a potential limitation. 
8. Page 10: Again, related to the issue with Phase 2 including only CISS students, the authors stated that because the pilot course was used to pilot the content of the course rather than the multidisciplinary aspects, that “ *the validity of the results is unaffected by this.*” Please clarify this statement. Also consider whether this should be listed as a limitation in this design process.

Have any limitations of the research been acknowledged?

Yes

Is the work clearly and accurately presented and does it cite the current literature?

Yes

If applicable, is the statistical analysis and its interpretation appropriate?

Not applicable

Are all the source data underlying the results available to ensure full reproducibility?

Yes

Is the study design appropriate and is the work technically sound?

Yes

Are the conclusions drawn adequately supported by the results?

Yes

Are sufficient details of methods and analysis provided to allow replication by others?

Yes

Reviewer Expertise:

I have a PhD in Health Professions Education and have experience in curricular design and evaluation. My areas of research are in patient-provider communication and shared decision making.

I confirm that I have read this submission and believe that I have an appropriate level of expertise to confirm that it is of an acceptable scientific standard, however I have significant reservations, as outlined above.

# Response

**Published:** 2024-11-26

**Authors:** Jessica Oudenampsen

**Affiliations:** Radboudumc, Radboud Universiteit, Nijmegen, Gelderland, The Netherlands

Dear reviewer, 

 We appreciate the effort and the time you put into reviewing this manuscript. 

 In response to your comments, please see the table below. 

**Reviewer comment: **16. The authors utilized a design-based educational research approach to construct an interdisciplinary course in healthcare communications and share the iterative design process in this article. They used design principles to guide the construction, based on new theoretical and empirical knowledge. The work is clearly and accurately presented and cites some of the current literature, although the supporting references are not comprehensive.

**Response: **We thank the reviewer for the valuable feedback.

 The comments helped us considerably in strengthening and finetuning the manuscript.

 In response to this and other comments, we added several references throughout the manuscript.  

**Reviewer comment: **17. Page 3, second paragraph, the authors state: “Three categories of interdisciplinary learning outcomes can be distinguished: “(1) academic and disciplinary grounding, (2) metacognitive skills such as communication, creativity and critical thinking, and (3) perspective and perception change” and cite an unpublished paper by one of the authors (Oudenampsen *et al*, 2023 ^b^). Especially given the unpublished nature of the citation, would recommend a little more detail about these three categories. How were they conceived? If the unpublished paper contains research around these categories, would recommend more information about this research.

**Response: **We added more detail about the three categories, by explaining how the categories were defined:

- P.4: we added a description about academic and disciplinary grounding.
- p.4: we added examples of metacognitive skills
- p.4+5: we added a description about perspective changing.

The systematic review was conducted parallel to the current paper and is now published online (in the Review of Higher Education, https://doi.org/10.1353/rhe.2024.a930107). We chose not to describe the systematic review research process in the current manuscript for several reasons;

- Currently, the systematic review is published in the review of higher education, so the entire research method is available for consultation.
- The methods used in conducting a systematic review are comprehensive and require a detailed description. A brief description of the research methods in this paper will not extend beyond the general principles of conducting a systematic review. Assuming that the reader is familiar with the basis principles of a systematic review, we think a brief description of the methods used will not add any relevant information to the current manuscript. p. 4+5

**Reviewer comment: **18. The authors clearly state their objective in the last paragraph of the Introduction. This flows well from the background provided in the Introduction and helps frame the paper that follows.

**Response: ** Thank you, we are happy to hear this. Please note that in the revised version of the manuscript, we added some information to the introduction, e.g., what is interdisciplinary education and constructive alignment in interdisciplinary education. Furthermore, we rewrote the part that is describing the gap in the current literature. From our perspective, this makes sure that the objective flows even better from the background provided in the introduction. p. 4-8 19.

**Reviewer comment: **Page 3, Context: It might be helpful to provide more details about the undergraduate communication and information sciences (CISS) program. Because this program may not be familiar to many readers, it would be helpful to better understand the context of this study and how it may be similar or not to another context.

**Response: ** We thank the reviewer for this feedback. We added an appendix which provides an overview of the undergraduate program of communication and information sciences. The appendix is included in the dataset associated with this manuscript ( https://doi.org/10.34973/m7gm-8w65) (The appendix is not included in the manuscript according to the guidelines of MedEdPublish). Appendix C 20.

**Reviewer comment: **Page 3, Design: Would recommend more comprehensively addressing reflexivity, by providing, at the least, more details about the design group. 

**Response: **Thank you. We added more details about the design group. p. 6

**Reviewer comment: **21. Page 3, Phase 2: The authors state: “In the second design cycle, we piloted the content of the first version of the interdisciplinary course. Only CISS participated . . .”. It is not clear why this decision was made, given that the course objectives were likely centered around the interdisciplinary nature of the course—how were the authors able to “evaluate” the interdisciplinary objectives when it was only a single discipline who was participating?

**Response: ** Due to practical reasons, it was not possible to include medicine students to the first pilot of the course. We decided not to focus on evaluating the interdisciplinary aspects of the course in this pilot in the second design cycle but rather focused on evaluating the content of the course. We were able to evaluate the qualification aspects of the course (Biesta, 2010), for example if the content of the lectures provided enough (practical) information to be able to conduct a simulation conversation, and if the lectures provided CISS students with enough knowledge about the disease the simulation patient was suffering from. We concluded from this pilot study that the interdisciplinary aspect was missing, and that CIS students would benefit from adding medicine students and making it really interdisciplinary. We made several adjustments in the text to make this clearer.  p.7 & p.15

**Reviewer comment: **22. Page 4, Box 1: Why did the authors not include the course learning objectives in Box 1? Especially given that the authors did not find clearly delineated outcomes of interdisciplinary learning during the literature review in Phase 1, it would be important for the reader to understand what the design team developed for learning objectives.

**Response: ** We thank the reviewer for this feedback. We had not noticed that the learning objectives have dropped out of the box. We added them in the revised version of the manuscript. P. 10

**Reviewer comment: **23. Page 7, It seems that during Phase 2, changes were made to the content of the course based on the CISS students. Is this the case and, if so, was the design team concerned about this biasing the ultimate content toward what is needed for CISS students? While the authors state that the “pilot study confirmed the added value of the interdisciplinary aspect”, it seems important to acknowledge this as a potential limitation. 

**Response: **We indeed changed some relatively minor aspects of the course toward what was needed for CISS students. For example, we added a glossary to the course content about relevant terminology from both CIS and medicine. We also added practical examples from both the medicine as well as the CIS discipline to make clear to students why lectures were relevant to them and their future career.

 We added to the limitations section that changes made to the course in order to ensure it fitted the background knowledge of CISS may have biased the content of the course. p. 22

**Reviewer comment: **24. Page 10: Again, related to the issue with Phase 2 including only CISS students, the authors stated that because the pilot course was used to pilot the content of the course rather than the multidisciplinary aspects, that “the validity of the results is unaffected by this.” Please clarify this statement. Also consider whether this should be listed as a limitation in this design process.  

**Response: **We reframed the section about this limitation and added the limitation that was mentioned in the feedback above. p.22

# Reviewer report: Reviewer response for version 1

**DOI:** 10.21956/mep.21094.r34401

**Published:** 2023-09-07

**Authors:** Renate Klaassen

**Affiliations:** 1 TU Delft, Delft, Netherlands Antilles

This article uses a design-based research approach in which iterative evaluations have been implemented in an interdisciplinary learning (challenge) education. The aim was to improve the curricular design. Authors claim to have done a systemic or narrative review of the literature; however, they have found few relevant sources.

 Although the authors have done an enormous amount of work, which is appreciated, it does, in the 1st place, not seem to be an interdisciplinary course but rather a genre-specific linguistic/communicative analysis of practice in a medical environment. The argument might be that it became more interdisciplinary after the pilot as different types of students participated. However, it is still not clear how the interdisciplinary is realized. That touches upon the other issues. This article is very applied and practice-focused and lacks scientific rigour, as well, at times, rather presumptuous.

 The Narrative and Systemic literature review provide minimal theoretical grounding as a dismal number of consulted literature is mentioned. The argument that a lack of frameworks is somewhat unsubstantiated; consult Boon, Repko, Dorst, Keestra & de Greeff, Latucca, and many more. Coming up with “the seven design principles” seems a bit arrogant, as is the idea that the authors were the first interdisciplinary course in this field. Moreover, the scientific methods of evaluations are unclear. It seems not more than a generic course evaluation without any specific variables that have been questioned. Indeed, a clear problem definition and research question are lacking. So, it is hard to assess the credibility & validity of the claims made in this article. 

 It probably would be better if it were published in a journal of applied practice.

Have any limitations of the research been acknowledged?

Partly

Is the work clearly and accurately presented and does it cite the current literature?

Partly

If applicable, is the statistical analysis and its interpretation appropriate?

Not applicable

Are all the source data underlying the results available to ensure full reproducibility?

No

Is the study design appropriate and is the work technically sound?

Partly

Are the conclusions drawn adequately supported by the results?

Partly

Are sufficient details of methods and analysis provided to allow replication by others?

Partly

Reviewer Expertise:

Interdisciplinary Education, English medium instruction, Engineering Education

I confirm that I have read this submission and believe that I have an appropriate level of expertise to state that I do not consider it to be of an acceptable scientific standard, for reasons outlined above.

# Response

**Published:** 2024-11-26

**Authors:** Jessica Oudenampsen

**Affiliations:** Radboudumc, Radboud Universiteit, Nijmegen, Gelderland, The Netherlands

Dear reviewer, 

 We appreciate the effort and the time you put into reviewing this manuscript. 

 In response to your comments, please see the table below. 

**Reviewer comments: **14. This article uses a design-based research approach in which iterative evaluations have been implemented in an interdisciplinary learning (challenge) education. The aim was to improve the curricular design. Authors claim to have done a systemic or narrative review of the literature; however, they have found few relevant sources.

**Reviewer comments: **Although the authors have done an enormous amount of work, which is appreciated, it does, in the 1st place, not seem to be an interdisciplinary course but rather a genre-specific linguistic/communicative analysis of practice in a medical environment. The argument might be that it became more interdisciplinary after the pilot as different types of students participated. However, it is still not clear how the interdisciplinary is realized. That touches upon the other issues. This article is very applied and practice-focused and lacks scientific rigour, as well, at times, rather presumptuous.

**Response: **We thank the reviewer for the detailed feedback to the manuscript.

 In the following part we will respond to the feedback and indicate the changes we made.

- We added information about the design of Phase 1 (literature review).
- We clarified that the systematic review we cite pertains to the learning outcomes of interdisciplinary education, published elsewhere (in the Review of Higher Education).

We thank the reviewer for the critical feedback on the interdisciplinarity aspects of the course. From our perspective, the course is interdisciplinary. Indeed, the pilot course in which only CIS students participated, was not interdisciplinary. We mention this in the manuscript on p. 22.

 We further elucidated the interdisciplinary nature of the actual course (not the pilot). During the course we paid explicit attention to dialogue among students of different disciplines, focusing on key interdisciplinary learning outcomes (see Oudenampsen et al., 2024) such as disciplinary grounding and perspective taking. Students worked together in interdisciplinary groups to analyze different doctor-patient conversations. In doing so, they needed to integrate medical perspectives, e.g., about content of medical conversations and conversation skills, and communication perspectives, e.g., about strategies in resistance and influencing, into a scientific report on communication and influencing in medical communication practice.

 To further clarify how interdisciplinarity was realized, we made some adjustments to several parts of the manuscript:

- We added the educational formats and learning activities to box 1. P. 7, P. 14, P. 22, P. 6, P. 8-10 15.

**Reviewer comments: **The Narrative and Systemic literature review provide minimal theoretical grounding as a dismal number of consulted literature is mentioned. The argument that a lack of frameworks is somewhat unsubstantiated; consult Boon, Repko, Dorst, Keestra & de Greeff, Latucca, and many more. Coming up with “the seven design principles” seems a bit arrogant, as is the idea that the authors were the first interdisciplinary course in this field. Moreover, the scientific methods of evaluations are unclear. It seems not more than a generic course evaluation without any specific variables that have been questioned. Indeed, a clear problem definition and research question are lacking. So, it is hard to assess the credibility & validity of the claims made in this article. 

 It probably would be better if it were published in a journal of applied practice.

**Response: **Thank you for these comments, which helped us to rephrase our goals in the following ways: - We clarified that the systematic review we refer to is published elsewhere. - We deleted the sentence that was claiming that a framework with designable elements for interdisciplinary learning is still lacking. We added several sentences to the respective paragraph to clarify that, indeed, other authors described design principles, and that the added value of the design principles described in our manuscript lies in the fact that it is based on an iterative education process and on empirical research about to this education module.   -We also clarified that this is not the first paper describing a course in communication for medicine students, however, most previous papers are about monodisciplinary or interprofessional and not interdisciplinary education.

 Please see also our comments to #8 and #10 (reviewer 1) and #14. We explain in the paper that we proceeded along several lines to evaluate the interdisciplinary course: First, we combined the generic course evaluations of the three participating faculties to allow for an interdisciplinary course evaluation. Second, we included specific questions about interdisciplinary teaching, based on the literature of interdisciplinary learning outcomes. Third, the evaluation as well as the results of all the evaluations in different phases are included in the extensive dataset that is associated with this manuscript (and of which we added the reference(s) to the manuscript). Fourth, the information from focus groups, individual interviews and panel discussions formed a solid evaluation basis. These findings are described elsewhere (please see refs in the manuscript). P. 9, P. 5, P. 5

# Reviewer report: Reviewer response for version 1

**DOI:** 10.21956/mep.21094.r34516

**Published:** 2023-08-31

**Authors:** Hester Smeets

**Affiliations:** 1 Zuyd University of Applied Sciences, Heerlen, Netherlands Antilles

This manuscript provides insight in a very complex topic, namely interprofessional education (IPE); many educators struggle with the design of IPE and interprofessional (IP) assessment. I believe that design-based research is a very suitable approach to provide us with more insights into how and especially why certain educational innovations work (and which don't). 

 Though I think this topic is of great importance, I have several questions regarding this manuscript. 

 A key question I have with this manuscript is why you chose to publish this as a manuscript - it feels as if multiple sub-studies are combined in this manuscript, leading to little information about a lot. Perhaps it's better to turn this around, and to write a lot of detail about a somewhat smaller study. 

 A very linguistic question would be why you chose interdisciplinary instead of interprofessional? Most of the key literature (e.g. Frenk et al., 2010; WHO, 2010) use the term interprofessional education. 

**Introduction**

 The introduction touches upon some of the relevant reasons to implement IPE. 

 However, I think the introduction went a little bit too fast from IP collaboration - IPE to your methods. I missed the aim of this manuscript; is it the description of? And how could you distill design guidelines from a description? 

 I'm missing some important (up to date) literature in the introduction. I believe the definition for IPE you use is quite old, and I think the field has gained a lot of attention in the last years, so the definition has changed as well. 

 The categories of IPE you describe require more explanation for me. 

 You write about a systematic approach, and a framework. It's not clear to me why we need this after reading, and I was wondering whether the approach is the same as the framework? 

**Methods**

 I believe that the methods section is missing important information to be able to replicate this research. For instance, you speak about a narrative review, but after reading, I don't know how how the review was conducted - where did you look for the articles? Which articles did you include? What was the research question, etc. 

 Regarding the course description, I believe that a visual aid would help the reader here. It's a lot of information, and assessment is quite torn apart from education here. It would help to make this visual so the reader instantly knows which assessment was performed when and by whom, etc. 

 Figure 1 is helpful, but it provides a lot of information. Through the methods section, I miss important information regarding participants, materials used (for the research, NOT for the education), the research procedure, the analysis, the trustworthiness. 

 What were the research questions you were looking to answer? 

**Results**

 The results section for phase 1 does not seem to match with the methods section of phase 1 - it seems that you explain your own curriculum, whereas in the methods, you explain that you conducted a review of the literature. Since communication is an important aspect of IPE, were there no articles about this in the literature? What search terms did you use, and where did you search? Which time frame? I googled the topics and I found some articles, but of course I'm not sure what you were specifically looking at. 

 Phase 2 results - this sounds a bit like the procedure section which I would expect in the methods. It is unclear to me how you analyzed which data from whom. 

 Same goes for phase 3. 

 I would suggest to publish this paper as an example of an interprofessional education course. To present guidelines, I would expect more details about the methods as to how you distilled the guidelines from the data you gathered in this very extensive process.

Have any limitations of the research been acknowledged?

Yes

Is the work clearly and accurately presented and does it cite the current literature?

Partly

If applicable, is the statistical analysis and its interpretation appropriate?

Not applicable

Are all the source data underlying the results available to ensure full reproducibility?

No

Is the study design appropriate and is the work technically sound?

Yes

Are the conclusions drawn adequately supported by the results?

Partly

Are sufficient details of methods and analysis provided to allow replication by others?

No

Reviewer Expertise:

Interprofessional education, assessment in interprofessional education, design-based research, qualitative research

I confirm that I have read this submission and believe that I have an appropriate level of expertise to state that I do not consider it to be of an acceptable scientific standard, for reasons outlined above.

## References

[1] : Health professionals for a new century: transforming education to strengthen health systems in an interdependent world. *The Lancet* .2010;376(9756) : 10.1016/S0140-6736(10)61854-5 1923-1958 10.1016/S0140-6736(10)61854-5 21112623

[2] : Framework for action on interprofessional education.2010;

# Response

**Published:** 2024-11-26

**Authors:** Jessica Oudenampsen

**Affiliations:** Radboudumc, Radboud Universiteit, Nijmegen, Gelderland, The Netherlands

Dear reviewer, 

 We appreciate the effort and the time that you put into reviewing this manuscript. 

 In response to your comments, please see the table below. 

**Editor’s and Reviewer’s Comments** **Our Response** **Location of edits** **Reviewer #1**

**Reviewer’s Comments: **1. This manuscript provides insight in a very complex topic, namely interprofessional education (IPE); many educators struggle with the design of IPE and interprofessional (IP) assessment. I believe that design-based research is a very suitable approach to provide us with more insights into how and especially why certain educational innovations work (and which don't). 

**Response: **Thank you, we appreciate the positive comments. To be clear, this manuscript provides insights in *interdisciplinary* education (IDE) instead of interprofessional education (IPE). To elucidate how IDE differs from IPE, we added a paragraph to the introduction section: ‘What is interdisciplinary education’. The definitions correspond to as it is more often used in higher education and in literature (Repko and Szostak, 2020), such as at Utrecht University (see also: https://edusources.nl/materials/133432c1-f600-42fd-96f3-9eea4f22965f/interdisciplinarity-and-interprofessionality-explained ). P. 4-5

**Reviewer’s Comments: **2. A key question I have with this manuscript is why you chose to publish this as a manuscript - it feels as if multiple sub-studies are combined in this manuscript, leading to little information about a lot. Perhaps it's better to turn this around, and to write a lot of detail about a somewhat smaller study. 

**Response: ** We chose the genre of a research-based paper outlining design principles for interdisciplinary education, because more insights into design principes would be a significant contribution to the field. Currently, such articles are (almost) nonexistent. We therefore believe that an integrated representation and analysis of the iterative design process of an interdisciplinary course, as in the current manuscript, offers an added value to the research field.        

**Reviewer’s Comments: **3. A very linguistic question would be why you chose interdisciplinary instead of interprofessional? Most of the key literature (e.g. Frenk et al., 2010; WHO, 2010) use the term interprofessional education. 

**Response: **Please see our answer to #1. We added a paragraph to the introduction called ‘What is interdisciplinary education’ to further clarify how this is, in our opinion, not a linguistic question, but a different educational concept. P. 4-5 4

**Reviewer’s Comments: ** The introduction touches upon some of the relevant reasons to implement IPE. 

 However, I think the introduction went a little bit too fast from IP collaboration - IPE to your methods. I missed the aim of this manuscript; is it the description of? And how could you distill design guidelines from a description? 

**Response: **We made some changes to the introduction section in response to this feedback:

- We added a paragraph about constructive alignment in interdisciplinary education, which builds towards the goal of this manuscript
- We added some sentences on the gap in literature on interdisciplinary education
- We reframed the aim of the manuscript in the last paragraph of the introduction, for further clarification
- We added that we provide further background information in part 1 of the results of the manuscript. P. 4, P. 5, P. 5, P. 6 5.

**Reviewer’s Comments: ** I'm missing some important (up to date) literature in the introduction. I believe the definition for IPE you use is quite old, and I think the field has gained a lot of attention in the last years, so the definition has changed as well. 

**Response: **We used the definition of interdisciplinary education as is most referred to in literature on interdisciplinarity. As stated before, from our perspective interprofessional and interdisciplinary education are two different concepts. Obtaining relevant literature is part of the design- based approach in phase 1. At the end of the introduction, we therefore added a sentence that clarifies that a more comprehensive overview of existing literature about (the design of) interdisciplinary education is provided in the results section 1. P. 6 6.

**Reviewer’s Comments: ** The categories of IPE you describe require more explanation for me. 

**Response: **It is not quite clear which categories the reviewer refers to. Assuming that the reviewer refers to the categories in which learning outcomes of interdisciplinary education can be divided, we made several adjustments.

- P.4: we added a description about academic and disciplinary grounding.
- p.4: we added examples of metacognitive skills
- p.4+5: we added a description about perspective changing. P. 6+7 7. 

**Reviewer’s Comments: **You write about a systematic approach, and a framework. It's not clear to me why we need this after reading, and I was wondering whether the approach is the same as the framework? 

**Response: **We applied several changes in this paragraph in the revised manuscript. We believe this strengthened this part of the manuscript.  

- We elaborated on interdisciplinary education to make clear in which perspective the current research was conducted.
- We deleted the sentences that contained the mentioned parts with ‘systematic approach’ and ‘framework’ and rewrote it.
- We added a gap and the goal of the current research in the introduction section. P. 4 + 5 8.

**Reviewer’s Comments:**  I believe that the methods section is missing important information to be able to replicate this research. For instance, you speak about a narrative review, but after reading, I don't know how the review was conducted - where did you look for the articles? Which articles did you include? What was the research question, etc. 

**Response: **We appreciate the feedback on the methods section. We deleted the term narrative review to avoid confusion about the procedure we used.

 We added some information to the methods section:

- We added the research question: What are the elements in education that are necessary to achieve interdisciplinary learning in students in interdisciplinary education.
- We added in which databases we searched. P. 7   P. 7 9. 

**Reviewer’s Comments:**  Regarding the course description, I believe that a visual aid would help the reader here. It's a lot of information, and assessment is quite torn apart from education here. It would help to make this visual so the reader instantly knows which assessment was performed when and by whom, etc. 

**Response: **We thank the reviewer for suggesting this idea. We added a visual that clarifies the correlations between educational content and assessment with constructive alignment. Figure A,  P.11 10.

**Reviewer’s Comments:** Figure 1 is helpful, but it provides a lot of information. Through the methods section, I miss important information regarding participants, materials used (for the research, NOT for the education), the research procedure, the analysis, the trustworthiness. 

 What were the research questions you were looking to answer? 

**Response: **We thank the reviewer for this feedback.

 In response to this feedback, we added information regarding several aspects in the methods as well as in the results section to elucidate the principles of a design-based article and relevant steps taken in this approach:

- We added additional information about the literature search (see also comment #8)
- We added the fact that we conducted an in-class evaluation with the participants of the harmonic initiative during the last session of this educational initiative.
- We added that we conducted a team meeting with evaluation with all involved teachers in the harmonic initiative
- We added information about the source of the conclusions drawn in phase 2.
- We added a description of the course evaluations in phase 3, as well as links to the dataset and relevant descriptions of methods of the evaluation(s) and focus groups and interviews.
- We added data about the evaluations in the second design cycle (e.g., participant numbers). - We added data about the course evaluations in the third design cycle (e.g. participant numbers, spread between disciplines, dataset link). P. 7, P. 7, P. 7, p.14 + 15, P. 7, P. 15, P. 16+ 17 11.

**Reviewer’s Comments: **The results section for phase 1 does not seem to match with the methods section of phase 1 - it seems that you explain your own curriculum, whereas in the methods, you explain that you conducted a review of the literature. Since communication is an important aspect of IPE, were there no articles about this in the literature? What search terms did you use, and where did you search? Which time frame? I googled the topics, and I found some articles, but of course I'm not sure what you were specifically looking at. 

**Response: **Please also see our response to comments #1 and #8. The aim of the analysis and exploration phase in educational design research is to seek out and learn from how others have viewed and attended to similar problems. We therefore conducted a comprehensive literature search to find previous literature about design principles for interdisciplinary education. We then applied these literature findings to our own curriculum.

 We made some adjustments to the text in the manuscript to clarify this process of the first research phase.   We did not include research on communication. Communication is an important aspect of IPE and IDE. However, in most literature, communication is practiced from a strictly medical perspective, which is not relevant for the topic of interdisciplinary communication education, i.e., education that involved students from (at least) two different disciplines. As far as we know, papers that reviewed such a program, and/or the design principles of such a program have not been published thus far. P. 7 12.

**Reviewer’s Comments: ** Phase 2 results - this sounds a bit like the procedure section which I would expect in the methods. It is unclear to me how you analyzed which data from whom. 

 Same goes for phase 3. 

**Response: **We rearranged several aspects of the manuscript:

- We added more details about the procedure in the methods section, for both phase 2 and phase 3. P. 7 13.

**Reviewer’s Comments: **​​​​​​​ I would suggest to publish this paper as an example of an interprofessional education course. To present guidelines, I would expect more details about the methods as to how you distilled the guidelines from the data you gathered in this very extensive process.

**Response: **We believe that the modifications we made in the manuscript, as well as the references to the extensive dataset(s) associated to this research, have improved the manuscript to such an extent that the reader is convinced of the guidelines we provide here.
